# Diagnostic Laboratory Features and Performance of an *Aspergillus* IgG Lateral Flow Assay in a Chronic Pulmonary Aspergillosis Cohort

**DOI:** 10.1128/spectrum.00264-23

**Published:** 2023-05-01

**Authors:** Rong-Sheng Zhu, Ling-Hong Zhou, Jia-Hui Cheng, Yu Luo, Wen-Jia Qiu, Jun-Tian Huang, Ying-Kui Jiang, Hua-Zhen Zhao, Xuan Wang, Zhong-Qing Chen, Li-Ping Zhu

**Affiliations:** a Department of Infectious Diseases, Shanghai Key Laboratory of Infectious Diseases and Biosafety Emergency Response, National Medical Center for Infectious Diseases, Huashan Hospital, Fudan University, Shanghai, China; b Department of Pathology, Huashan Hospital, Fudan University, Shanghai, China; Universidade de Brasilia

**Keywords:** chronic pulmonary aspergillosis, diagnostics, lateral flow assay, *Aspergillus* IgG antibody, serology

## Abstract

Chronic pulmonary aspergillosis (CPA) is a chronic and progressive fungal disease with high morbidity and mortality. Avoiding diagnostic delay and misdiagnosis are concerns for CPA patients. However, diagnostic practice is poorly evaluated, especially in resource-constrained areas where Aspergillus antibody testing tools are lacking. This study aimed to investigate the diagnostic laboratory findings in a retrospective CPA cohort and to evaluate the performance of a novel Aspergillus IgG lateral flow assay (LFA; Era Biology, Tianjin, China). During January 2016 and December 2021, suspected CPA patients were screened at the Center for Infectious Diseases at Huashan Hospital. A total of 126 CPA patients were enrolled. Aspergillus IgG was positive in 72.1% with chronic cavitary pulmonary aspergillosis, 75.0% with chronic necrotizing pulmonary aspergillosis, 41.7% with simple aspergilloma, and 30.3% with Aspergillus nodule(s). The cavitary CPA subtypes had significantly higher levels of Aspergillus IgG. Aspergillus IgG was negative in 52 patients, who were finally diagnosed by histopathology, respiratory culture, and metagenomic next-generation sequencing (mNGS). Sputum culture was positive in 39.3% (42/107) of patients and Aspergillus fumigatus was the most common species (69.0%, 29/42). For CPA cohort versus controls, the sensitivity and specificity of the LFA were 55.6% and 92.7%, respectively. In a subgroup analysis, the LFA was highly sensitive for A. fumigatus-associated chronic cavitary pulmonary aspergillosis (CCPA; 96.2%, 26/27). Given the complexity of the disease, a combination of serological and non-serological tests should be considered to avoid misdiagnosis of CPA. The novel LFA has a satisfactory performance and allows earlier screening and diagnosis of CPA patients.

**IMPORTANCE** There are concerns on avoiding diagnostic delay and misdiagnosis for chronic pulmonary aspergillosis due to its high morbidity and mortality. A proportion of CPA patients test negative for Aspergillus IgG. An optimal diagnostic strategy for CPA requires in-depth investigation based on real-world diagnostic practice, which has been rarely discussed. We summarized the clinical and diagnostic laboratory findings of 126 CPA patients with various CPA subtypes. Aspergillus IgG was the most sensitive test for diagnosing CPA. However, it was negative in 52 patients, who were finally diagnosed by non-serological tests, including biopsy, respiratory culture, and metagenomic next-generation sequencing. We also evaluated a novel Aspergillus IgG lateral flow assay, which showed a satisfactory performance in cavitary CPA patients and was highly specific to Aspergillus fumigatus. This study gives a full picture of the diagnostic practice for CPA patients in Chinese context and calls for early diagnosis of CPA with combined approaches.

## INTRODUCTION

Chronic pulmonary aspergillosis (CPA), first systemically recognized in early 1980s (1, 2), is a chronic lung infection caused by Aspergillus species. In past decades, CPA has gained increasing clinical attention and is recognized as a potential public health threat. The disease burden of CPA is estimated to be over 3,000,000 cases worldwide, and approximately one-third of patients have it as a sequel to pulmonary tuberculosis (PTB) (3, 4). Patients can manifest with overlapping forms over the course of the disease. The 5-year survival rate is around 50% to 85% (5). Optimal diagnostic methods for early recognition of CPA are urgently needed.

The diagnosis of CPA requires a combination of compatible symptoms, radiological abnormalities, and microbiological evidence. There are a series of serological and non-serological tests to confirm Aspergillus infection. Currently, Aspergillus IgG antibody testing plays a central role in CPA diagnosis, and the most widely used method is enzyme-linked immunosorbent assay (ELISA) (6). The sensitivity and specificity of mainstream commercial ELISAs range from 86% to 98% and 90% to 99%, respectively (6, 7). The optimal cutoff values for these assays in different populations are mostly undetermined. Notably, negative Aspergillus IgG antibody test results associated with non-*fumigatus* Aspergillus infection, immunodeficiency, and indolent CPA phenotypes have been noted (8). A combined approach to CPA diagnosis is warranted (9).

**FIG 1:**
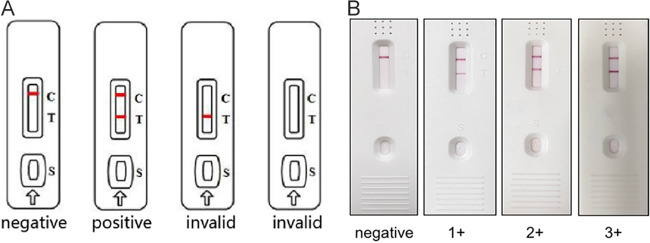
Illustrative (A) and representative (B) results of the Aspergillus IgG lateral flow assay.

Globally, CPA is still under-recognized because current commercial Aspergillus IgG assays are often not accessible or cost-effective in many resource-constrained areas, where direct microscopy and fungal culture are alternative, low-sensitivity methods of CPA diagnosis. In recent years, immunochromatography has been used to detect Aspergillus-specific antibodies in the form of a lateral flow assay (LFA). LFA has the advantages of simplicity, speed, and cost-effectiveness over ELISA in identifying CPA patients (10). In this study, we performed a comprehensive analysis on the diagnostic laboratory findings of CPA patients in a Chinese context to explore an optimal strategy to identify the disease early and accurately. In addition, we evaluated the performance of a novel Aspergillus IgG LFA (Era Biology, Tianjin, China) in our CPA cohort. The illustrative and representative examples of the LFA were shown in Fig. 1.

## RESULTS

### Patient characteristics.

**FIG 2:**
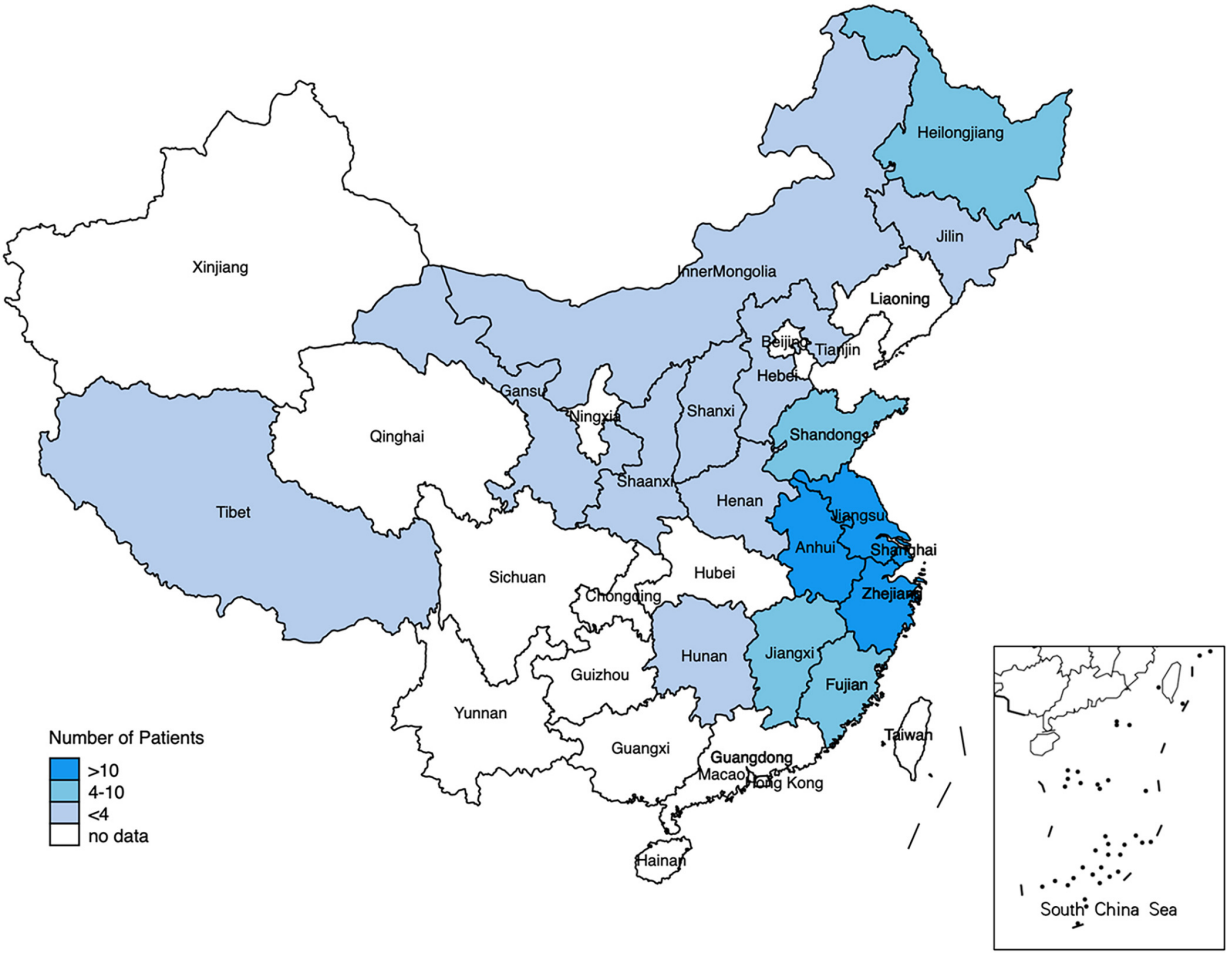
Geographic distribution of chronic pulmonary aspergillosis (CPA) patients, 2016 to 2021.

A total of 126 CPA patients were enrolled in this study, including 62 proven and 64 probable cases, during January 2016 and December 2021. The geographic distribution of the CPA patients is illustrated in Fig. 2. Patients were referred from across the nation and distributed across most climate zones in the mainland.

Demographic and clinical characteristics of CPA patients are shown in Table 1. The median age of disease onset was 57 (50 to 65) years, with male dominance. Underlying conditions were identified in most patients (91.5%), and pulmonary tuberculosis (31.7%) was the most common underlying disease for CPA development. The most common symptoms were cough (76.1%) and hemoptysis (50.0%), followed by fever (23.8%) and dyspnea (13.4%). Chronic cavitary pulmonary aspergillosis (CCPA) was the most common subtype (46.8%, 59/126), followed by Aspergillus nodules (AN; 26.2%, 33/126), chronic necrotizing pulmonary aspergillosis (CNPA; 15.9%, 20/126), simple aspergillosis (SA; 9.5%, 12/126), and chronic fibrosing pulmonary aspergillosis (CFPA; 1.6%, 2/126).

**TABLE 1:** Demographic and clinical characteristics of the CPA patients^*a*^

Variable	CPA patients (*n* = 126)
Age (yr), median (IQR)	57 (50–65)
Male, *n* (%)	79 (62.6)
BMI (kg/m^2^), median (IQR)	20.3 (18.0–23.8)
Clinical manifestations, *n* (%)	
Cough	96 (76.1)
Hemoptysis	63 (50.0)
Fever	30 (23.8)
Dyspnea	17 (13.4)
Fatigue	15 (11.9)
Chest pain	5 (3.9)
Underlying pulmonary disease, *n* (%)	
Pulmonary tuberculosis	40 (31.7)
Bronchiectasis	27 (21.4)
Bullae/emphysema	19 (15.0)
Previous thoracic surgery	15 (11.9)
COPD	14 (11.1)
Lung cancer history	7 (5.6)
NTM-PD	5 (4.0)
Comorbidities, *n* (%)	
Diabetes mellitus	21 (16.7)
Autoimmune diseases	21 (16.7)
Hypertension	19 (15.1)
Use of immunosuppressive agents, *n* (%)	21 (16.7)
CPA subtype, *n* (%)	
CCPA	59 (46.8)
AN	33 (26.2)
CNPA	20 (15.9)
SA	12 (9.5)
CFPA	2 (1.6)

a CPA, chronic pulmonary aspergillosis; IQR, interquartile range; BMI, body mass index; COPD, chronic obstructive pulmonary disease; NTM-PD, nontuberculous mycobacterial pulmonary disease; CCPA, chronic cavitary pulmonary aspergillosis; AN, Aspergillus nodule(s); CNPA, chronic necrotizing pulmonary aspergillosis; SA, simple aspergilloma; CFPA, chronic fibrosing pulmonary aspergillosis.

### Diagnostic features.

Diagnostic laboratory findings are detailed in Table 2. A total of 107 patients underwent sputum culture for fungi. The positive rate of culture was 39.3% (42/107), and A. fumigatus (69.0%, 29/42) was the most common species. Bronchoscopy was performed in 51 patients and bronchoalveolar lavage fluid (BALF) samples were routinely sent for fungal culture, with a positivity rate of 15.7% (8/51), which identified A. fumigatus in 5 patients, A. niger in 2 patients, and unspecified Aspergillus in 1 patient. PCR was positive for 21 patients by metagenomic next-generation sequencing (mNGS), and 6 of these also had compatible positive sputum culture results. Both mNGS and fungal culture identified A. fumigatus in 4 patients; the Aspergillus species in the remaining 2 patients were confirmed by mNGS and included A. niger and A. flavus, respectively. A total of 62 patients had histopathological findings suggestive of non-invasive Aspergillus hyphae through percutaneous/transbronchial lung biopsy and/or surgery. Most of these proven cases were referred from other hospitals. Notably, patients presenting with Aspergillus nodule(s) were common in our cohort, and 84.8% (28/33) were pathologically confirmed. For the remaining 5 probable cases, we observed responsiveness to antifungal treatment on follow-up.

**TABLE 2:** Diagnostic features of CPA patients^*a*^

Microbiological evidence	All CPA (*n* = 126)	CCPA^*b*^ (*n* = 61)	CNPA (*n* = 20)	SA (*n* = 12)	AN (*n* = 33)
Sputum culture positive, % (*n*/total)	39.3% (42/107)	45.9% (28/61)	65.0% (13/20)	0.0% (0/8)	5.6% (1/18)
A. fumigatus (*n*)	29	22	7	0	0
A. niger (*n*)	5	0	4	0	1
Aspergillus spp. (*n*)	8	6	2	0	0
BALF culture positive, % (*n*/total)	15.7% (8/51)	18.8% (6/32)	22.2% (2/9)	0.0% (0/3)	0.0% (0/7)
A. fumigatus (*n*)	5	5	0	0	0
A. niger (*n*)	2	0	2	0	0
Aspergillus spp. (*n*)	1	1	0	0	0
mNGS positive, n	21	11	5	2	3
A. fumigatus (*n*)	13	9	1	1	2
A. niger (*n*)	4	1	3	0	0
Other (*n*)	4	1^*d*^	1^*e*^	1^*e*^	1^*e*^
Histopathology positive, n	62	20	4	9	28
Percutaneous lung biopsy or TBLB (*n*)	19	11	2	1	5
Lung surgery (*n*)	43	10	2	8	23
Aspergillus IgG positive (IBL ELISA), % (*n*/total)	58.7% (74/126)	72.1% (44/61)	75.0% (15/20)	41.7% (5/12)	30.3% (10/33)
A. fumigatus-associated CPA^*c*^ (*n* = 39)	74.4% (29/39)	75.8% (22/29)	85.7% (6/7)	0.0% (0/1)	50.0% (1/2)
Non-*fumigatus* Aspergillus-associated CPA^*c*^ (*n* = 12)	66.7% (8/12)	50.0% (1/2)	62.5% (5/8)	0.0% (0/0)	100.0% (2/2)

a CPA, chronic pulmonary aspergillosis; CCPA, chronic cavitary pulmonary aspergillosis; CFPA, chronic fibrosing pulmonary aspergillosis; CNPA, chronic necrotizing pulmonary aspergillosis; SA, simple aspergilloma; AN, Aspergillus nodule; BALF, bronchoalveolar lavage fluid; mNGS, metagenomic next-generation sequencing; TBLB, transbronchial lung biopsy; ELISA, enyme-linked immunosorbent assay.

b Two CFPA patients were included.

c Aspergillus species confirmed by culture or mNGS.

d A. flavus was detected in one patient.

e Aspergillus spp. was detected in one patient.

**FIG 3:**
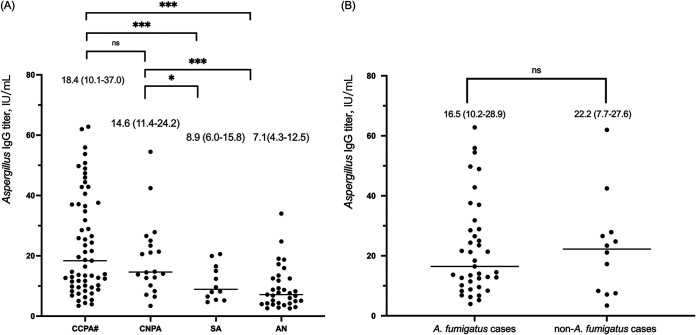
Distribution of Aspergillus IgG level measured by enzyme-linked immunosorbent assay (ELISA). Data are expressed as median and interquartile range. (A) Aspergillus IgG titers in different CPA subtypes. (B) Aspergillus IgG titers in A. fumigatus (*n* = 39) and non-A. fumigatus cases (10 A. niger and 2 A. flavus). Number sign (#) indicates that two chronic fibrosing pulmonary aspergillosis patients were included. CCPA, chronic cavitary pulmonary aspergillosis; CNPA, chronic necrotizing pulmonary aspergillosis; SA, simple aspergilloma; AN, Aspergillus nodule. ns, not significant; *, *P < *0.05; ***, *P < *0.001.

A total of 74 patients had positive Aspergillus IgG detected by the IBL ELISA (IBL International GmbH, Hamburg, Germany). The positivity rate was 72.1% in CCPA, 75.0% in CNPA, 41.7% in SA, and 30.3% in AN patients, as shown in Table 2. The CCPA and CNPA patients exhibited significantly higher median levels of Aspergillus IgG antibody than the SA and AN patients, as shown in Fig. 3A; while antibody levels were similar between A. fumigatus and non-A. fumigatus cases, as shown in Fig. 3B. Aspergillus IgG was negative in a total of 52 CPA patients, who were diagnosed by histopathology (*n* = 38), sputum fungal culture (*n* = 7), and mNGS (*n* = 7).

### Performance of the *Aspergillus* IgG LFA.

Control sera were obtained from 55 patients with proven lung diseases, including cryptococcosis (*n* = 27), mucormycosis (*n* = 9), mycobacterial lung disease (*n* = 5), lung cancer (*n* = 4), nocardiosis (*n* = 3), penicilliosis (*n* = 2), histoplasmosis (*n* = 2), chromomycosis (*n* = 1), coccidioidomycosis (*n* = 1), and actinomycosis (*n* = 1). The specificity of the Aspergillus IgG LFA was 92.7% (95% confidence interval [CI]: 82.4% to 98.0%). The diagnostic performance of the LFA is summarized in Table 3. The sensitivity, positive predictive value (PPV), negative predictive value (NPV), and Youden’s index in all CPA patients were 55.6% (95% CI: 46.4% to 64.4%), 0.95 (0.87 to 0.99), 0.48 (0.38 to 0.58), and 0.48 (0.29 to 0.62), respectively. In the subgroup analysis, the LFA was most efficient at diagnosing CCPA patients, with a sensitivity of 75.4% (95% CI: 62.7% to 85.5%). Regarding Aspergillus species, the LFA performed better for detecting A. fumigatus cases than non-A. fumigatus cases (87.2% versus 8.3%, *P* < 0.001).

**TABLE 3:** Diagnostic performance of the Aspergillus IgG LFA in CPA cohort^*a*^

Test group	Sensitivity, % (95% CI)	Specificity, % (95% CI)	PPV (95% CI)	NPV (95% CI)	Youden’s index (95% CI)
Non-CPA^*b*^ (*n* = 55)	-	92.7 (82.4–98.0)	-	-	-
CPA (*n* = 126)	55.6 (46.4–64.4)		0.95 (0.87–0.99)	0.48 (0.38–0.58)	0.48 (0.29–0.62)
A. fumigatus cases (*n* = 39)	87.2 (72.6–95.7)		0.89 (0.75–0.97)	0.91 (0.80–0.97)	0.80 (0.55–0.94)
non-A. fumigatus cases (*n* = 12)	8.3 (0.2–38.5)		0.20 (0.01–0.72)	0.82 (0.70–0.91)	0.01 (−0.17−0.36)
CCPA^*c*^ (*n* = 61)	75.4 (62.7–85.5)		0.92 (0.81–0.98)	0.77 (0.65–0.87)	0.68 (0.45–0.84)
CNPA (*n* = 20)	55.0 (31.5–76.9)		0.73 (0.45–0.92)	0.85 (0.73–0.93)	0.47 (0.14–0.75)
SA (*n* = 12)	25.0 (5.5–57.2)		0.43 (0.10–0.86)	0.85 (0.73–0.93)	0.18 (−0.12−0.55)
AN (*n* = 33)	30.3 (15.6–48.7)		0.71 (0.42–0.92)	0.69 (0.57–0.79)	0.23 (−0.02−0.47)

a LFA, lateral flow assay; CI, confidence interval; PPV, positive predictive value; NPV, negative predictive value; CPA, chronic pulmonary aspergillosis; CCPA, chronic cavitary pulmonary aspergillosis; CFPA, chronic fibrosing pulmonary aspergillosis; CNPA, chronic necrotizing pulmonary aspergillosis; SA, simple aspergilloma; AN, Aspergillus nodule.

b Non-CPA controls included diseased patients with cryptococcosis (*n* = 27), mucormycosis (*n* = 9), mycobacterial lung disease (*n* = 5), lung cancer (*n* = 4), nocardiosis (*n* = 3), penicilliosis (*n* = 2), histoplasmosis (*n* = 2), chromomycosis (*n* = 1), coccidioidomycosis (*n* = 1), and actinomycosis (*n* = 1).

c Two patients diagnosed with CFPA were included.

Patients with positive Aspergillus IgG LFA results showed significantly higher levels of antibody detected by the Aspergillus IgG ELISA than those with negative results (median level 14.1 versus 11.4; *P* < 0.001). Among the 126 patients enrolled in the cohort, positive Aspergillus IgG detected by ELISA was the only microbiological diagnostic finding in 10 patients. Table 4 shows a comparison of the performance of the LFA and ELISA in distinct CPA subtypes for the remaining 116 patients who were diagnosed with CPA through non-serological methods, including fungal culture, mNGS, and histopathology. The LFA demonstrated similar sensitivity to the ELISA for detecting Aspergillus IgG antibody (*P* = 0.895). Agreement between the LFA and ELISA was shown in 71.5% (83/116) of the patients, with a Cohen’s kappa coefficient of 0.426 (95% CI: 0.261 to 0.591).

**TABLE 4:** Comparison of the sensitivity and specificity of the Aspergillus IgG LFA and ELISA^*a*^

Parameter	LFA	ELISA	*P*
Specificity, % (*n*/total)			
Non-CPA^*b*^ (*n* = 55)	92.7 (51/55)	89.1 (49/55)	0.507
Sensitivity, % (*n*/total)			
CPA (*n* = 116)	54.3 (63/116)	55.2 (64/116)	0.895
A. fumigatus (*n* = 39)	87.2 (34/39)	74.4 (29/39)	0.151
non-A. fumigatus^*c*^ (*n* = 12)	8.3 (1/12)	66.7 (8/12)	0.009
CCPA^*d*^ (*n* = 53)	75.5 (40/53)	67.9 (36/53)	0.388
CNPA (*n* = 20)	55.0 (11/20)	75.0 (15/20)	0.185
SA (*n* = 11)	27.3 (3/11)	36.4 (4/11)	1.000
AN (*n* = 32)	28.1 (9/32)	28.1 (9/32)	1.000

a LFA, lateral flow assay; ELISA, enzyme-linked immunosorbent assay; CPA, chronic pulmonary aspergillosis; CCPA, chronic cavitary pulmonary aspergillosis; CNPA, chronic necrotizing pulmonary aspergillosis; SA, simple aspergilloma; AN, Aspergillus nodule. *P* was calculated using Pearson’s chi-squared test or Fisher’s exact test, as appropriate.

b Non-CPA controls included diseased patients with cryptococcosis (*n* = 27), mucormycosis (*n* = 9), mycobacterial lung disease (*n* = 5), lung cancer (*n* = 4), nocardiosis (*n* = 3), penicilliosis (*n* = 2), histoplasmosis (*n* = 2), chromomycosis (*n* = 1), coccidioidomycosis (*n* = 1), and actinomycosis (*n* = 1).

c Non-A. fumigatus cases included 2 A. flavus and 10 A. niger.

d Two patients diagnosed with chronic fibrosing pulmonary aspergillosis were included.

A total of 36 patients had inconsistent results. Of the 20 patients with positive ELISA/negative LFA results, 3 had Aspergillus IgG detected by the ELISA as the only microbiological evidence, while the remaining 17 had additional supportive laboratory findings, including fungal culture (*n* = 9), histopathology (*n* = 8), and mNGS (*n* = 3). Of the 16 patients with negative ELISA and positive LFA results, all were diagnosed by non-serological tests, including histopathology (*n* = 9), fungal culture (*n* = 5), and mNGS (*n* = 4).

## DISCUSSION

In this study, we comprehensively investigated the diagnostic laboratory findings of CPA patients in a Chinese context, among whom CCPA was the most common subtype and A. fumigatus was the most common causative species. Aspergillus IgG has proven to be reliable for establishing and/or confirming a CPA diagnosis compared with routine non-serological tests. In terms of simplicity, speed, and cost-effectiveness, the novel Aspergillus IgG LFA is deemed superior to the traditional ELISA for screening and earlier diagnosis of the disease, especially in resource-constrained settings (10).

The CPA patients in our cohort were referred from 17 provinces, showing a wide geographic distribution across mainland China. Considering that one-third of the patients had post-PTB sequalae, this distribution may have an epidemiological link with tuberculosis. Of the 40 patients with a history of PTB, 87.5% had residual cavities and 16.7% had received empirical antituberculosis treatment before their CPA diagnosis. China has the second largest predicted number of CPA patients based on the tuberculosis disease burden (3, 4). However, studies on CPA are much less reported from China due to the scarcity of clinical suspicion and diagnostic tools (15–17). CPA has a wide range of clinical and radiological features resulting from complex interactions between Aspergillus and its host (18), which can influence the sensitivity of different tests. Series work-ups should be performed to avoid misdiagnosis and delayed treatment.

Multiple tests are currently used for CPA diagnosis. Fungal culture from respiratory specimens is a well-established method and is widely used in clinical scenarios. The major limitation of culture is its low sensitivity, with an average reported positivity rate of 30%, as shown in our study. Moreover, Aspergillus is ubiquitously found in the environment and differentiating colonization from infection is challenging. Although galactomannan (GM) detection has been recognized as a key diagnostic test for invasive pulmonary aspergillosis (IPA) in neutropenic patients, its value for CPA diagnosis is still controversial even with BALF samples (19, 20). GM is often not present in blood in non-invasive Aspergillus infection and GM detection is not decisive enough to differentiate between CNPA and IPA, so it was not included as a supportive diagnostic element in this study. Most CPA patients have impaired lung function and persistent constitutional symptoms, so they often cannot tolerate procedures involving biopsy, surgery, and even endoscopy. A highly sensitive, discriminatory, and non-invasive method is thus of great importance.

Serum Aspergillus IgG can be detected in most cavitary CPA patients and has been defined as the core diagnostic element. Currently, Aspergillus IgG is mainly detected by ELISA, with sensitivity and specificity ranging from 70% to 96% and from 98% to 99%, respectively (6). The Aspergillus IgG ELISA in our cohort had a sensitivity of 66.6% (34/51) for CCPA and 75.0% (15/20) for CNPA and a specificity of 89.1% in the diseased controls. The novel Aspergillus IgG LFA, which uses colloidal gold immunochromatography, is portable and easy to use. When testing positive samples, the Aspergillus IgG antibody in the sample combines with gold-conjugated mouse anti-human IgG antibody to form an immune complex, which flows forward on the nitrocellulose membrane through the chromatography effect. In this study, we investigated its value for CPA diagnosis. The LFA showed a similar performance compared with the Aspergillus IgG ELISA, while it was more specific to A. fumigatus because it detected antibody in only 1 out of 12 patients with non-A. fumigatus infections. Because the antigen coated in the LFA test is purified thioredoxin reductase GliT from A. fumigatus, the LFA is expected to be less sensitive for detecting non-A. fumigatus cases, which are less frequently encountered in the CPA population. In contrast, the positive rate of Aspergillus IgG was not significantly different between A. fumigatus and non-A. fumigatus cases for the ELISA (29/39 versus 8/12, *P* = 0.715). The coated antigen from A. fumigatus in this assay is not disclosed. Of note, a substantial proportion of SA and AN patients were serum negative, which has also been observed in other studies using the ImmunoCAP system, a highly sensitive fluorescent enzyme immunoassay (21, 22). One study reported that in a cohort of pathologically confirmed cases of Aspergillus nodule(s), fewer than half (10/24) had positive Aspergillus IgG antibody (23). One possible explanation is that there is low disease activity in this subtype, with minor antibody production.

A. fumigatus is mainly responsible for CPA pathogenesis. However, the A. fumigatus antigens used in current commercial assays cannot produce a sufficient reaction with antibody induced by non-*fumigatus* Aspergillus species (8, 24), as is the case with the novel Aspergillus IgG LFA. As the most commonly used method, fungal culture should be performed regularly to identify potential non-*fumigatus* Aspergillus species, which allows the detection of antifungal resistance at the same time. Culture-independent molecular methods are alternatives to identify non-*fumigatus* Aspergillus (25). In recent years, mNGS has been rapidly applied for diagnosing invasive fungal infections (26). However, no data are available for its performance on CPA diagnosis. We identified 21 referred patients who were mNGS-positive for Aspergillus. The Aspergillus IgG positivity between mNGS-positive and culture-positive patients showed high concordance. The diagnostic performance of mNGS for CPA diagnosis requires further investigation.

In view of the favorable diagnostic performance of the Aspergillus IgG LFA in our CPA cohort, this assay complies with ASSURED (affordable, sensitive, specific, user-friendly, equipment-free, delivered) criteria (27). Currently, the only reported commercial LFA for Aspergillus antibody is produced by LDBio Diagnostics (Lyon, France). The sensitivity and specificity of the assay were 91.6% and 98.0%, respectively, in a French multicenter study (28), and these were validated in a UK cohort (7). The superior performance of the LDBio Aspergillus LFA may be because it detects both IgG and IgM in addition to different antigens used (mixed antigen from A. fumigatus). However, the assay showed less satisfactory performance in an Indonesian post-tuberculosis cohort, with sensitivity and specificity of 85.0% and 72.1%, respectively (29). A recent study in India also found that the sensitivity and specificity of the LDBio assay were 67.6% and 81%, respectively. Its performance was improved in patients with a history of tuberculosis (30). These discrepancies in different study populations impel more cohorts to validate the performance of the current Aspergillus IgG LFA.

Limitations were unavoidable in the current study. First, there is a potential bias in CPA patient enrollment. A total of 36 patients were serum negative by both Aspergillus IgG assays. Colonization could not be definitely excluded in patients with positive respiratory culture and/or mNGS results when treatment response on follow-up was unavailable. Second, half of the patients had been exposed to antifungals prior to admission, which may have impaired the sensitivity of Aspergillus IgG assays. However, this bias seemed to be negligible because the decline in Aspergillus IgG levels is often slow and most serum samples were collected within 6 months of the initial treatment. Third, this study was carried out in a single center, and most patients were referred from East China; a larger, multicenter study is needed to further evaluate the characteristics of CPA patients in China and validate the performance of the novel Aspergillus IgG LFA.

In conclusion, CPA is under-recognized and patients with cavitary pulmonary tuberculosis and chronic airway diseases are at high risk of CPA development. Multiple laboratory tests can aid in CPA diagnosis, among which Aspergillus IgG detection is the most sensitive and reliable. Regarding serum-negative patients, a combination of non-serological tests, including fungal culture, pathological investigation, and molecular method should be considered. The novel Aspergillus IgG LFA has satisfactory diagnostic performance and allows earlier diagnosis and efficient screening of CPA patients.

## MATERIALS AND METHODS

### Study design and population.

This study was carried out at the Center for Infectious Diseases, Huashan Hospital, Fudan University. During January 2016 and December 2021, patients who had persistent or progressive radiological abnormalities on a chest CT scan and presented clinical manifestations for at least 1 month were defined as suspected cases and were classified into proven and probable CPA cases based on the diagnostic criteria described below. Demographic data clinical and diagnostic laboratory findings of these patients were collected. After establishing the CPA cohort, we evaluated the performance of an Aspergillus IgG LFA (Era Biology, Tianjin, China). Diseased control sera were collected from patients with pathologically confirmed active lung diseases without evidence of Aspergillus infection or colonization. This study was reviewed and approved by the Huashan Hospital ethics review committee (approval no. 2022-1014).

### CPA diagnosis and classification.

The diagnosis of CPA was made according to the guidelines of the European Society for Clinical Microbiology and Infectious Diseases (ESCMID) and the European Respiratory Society (ERS) (11), with minor modifications. Patients with positive histopathology showing thin, hyaline, septate, acute-angle, and dichotomous branching hyphae compatible with Aspergillus spp. with or without positive culture from sterile lung tissue were defined as proven cases. Aspergillus hyphae did not invade the lung parenchyma except in cases of chronic necrotizing pulmonary aspergillosis. A probable diagnosis was made if the patient had any of the following evidence of Aspergillus infection: (i) positive Aspergillus DNA results in respiratory specimens, (ii) Aspergillus growth from respiratory culture, or (iii) positive Aspergillus IgG detected by an Aspergillus IgG ELISA (IBL International GmbH, Hamburg, Germany). Fungal culture and species identification were performed by the clinical mycology laboratory following standard operating procedures as previously described (12). Isolate information provided by the laboratory was based on phenotypic identification. Aspergillus DNA from respiratory samples was detected by a metagenomic next-generation sequencing technique following library construction and bioinformatic analysis through standard analytic workflows as described previously (13).

CPA patients were divided into five different subtypes based on clinical and radiological characteristics according to ESCMID guidelines (11): chronic cavitary pulmonary aspergillosis, chronic necrotizing pulmonary aspergillosis, chronic fibrosing pulmonary aspergillosis, simple aspergilloma, and Aspergillus nodule(s).

### *Aspergillus* IgG detection.

The Aspergillus IgG ELISA was performed according to the manufacturer’s instructions as described by Kim et al. (14). For the Aspergillus IgG LFA, 50 μL diluted serum sample (1:10 dilution) was added into the sample hole of the cassette. The result was read by the naked eye after 10 min. A red band in both the test (T) and control (C) lines indicated a positive result, while a red band only in the C line indicated a negative result. If there was no red band in the C line, the test was invalid and was repeated. In rare cases, an “equivocal” result was seen in which a very faint band appeared in the T line. This result was considered negative. Positive results ranged from weak positive (+) to strong positive (+++) based on the relative intensity of the C and T lines, see Fig. 1.

### Statistical analysis.

Continuous data were expressed as median and interquartile range (IQR) and were compared using a Mann–Whitney U test. For categorical variables, data were reported as numbers (percentages) and compared between groups using Pearson’s chi-squared test or Fisher’s exact test, as appropriate. Pairwise comparison of sensitivity levels between the Aspergillus IgG ELISA and LFA was performed using McNemar’s test. Diagnostic performance, including sensitivity, specificity, positive predictive value (PPV), negative predictive value (NPV) and Youden’s index of the LFA were calculated. *P* < 0.05 was considered statistically significant for all tests. Data were analyzed using Stata/SE version 15.0 software (StataCorp, College Station, TX), R statistical software version 4.1.3 (R Foundation for Statistical Computing) within RStudio version 1.4.1564 (RStudio), and Graph Pad Prism 9.0 (Graph Pad, San Diego, CA).

### Data availability.

The data that support the findings of this study are available on request from the corresponding authors. Data are not publicly available due to privacy and ethical restrictions.
